# Relation between Feed Particle Size Distribution and Plumage Condition in Laying Hens on Commercial Farms

**DOI:** 10.3390/ani11030773

**Published:** 2021-03-11

**Authors:** Ruben Schreiter, Klaus Damme, Markus Freick

**Affiliations:** 1 ZAFT e.V. Centre for Applied Research and Technology, D-01069 Dresden, Germany; markus.freick@htw-dresden.de; 2 Bavarian State Farms, Research and Education Center for Poultry, D-97318 Kitzingen, Germany; klaus.damme@baysg.bayern.de; 3 Faculty of Agriculture/Environment/Chemistry, HTW Dresden–University of Applied Sciences, D-01326 Dresden, Germany

**Keywords:** egg production, animal welfare, layers, feather pecking, sieve analysis

## Abstract

**Simple Summary:**

Feather pecking is a serious problem in laying hen husbandry, which can lead to feather damage, performance losses and economic disadvantages. In this context, feed has been identified as an important factor, whereby scientific knowledge is primarily available on the effect of ingredients but hardly on the feed structure. In this study, feed samples from feather pecking and non-feather pecking herds from production farms were analyzed for their nutrient contents and feed particle sizes. More coarser (>2.00 mm) and fewer medium and finer feed particles (≤1.60 mm) were found in the feed of the flocks affected by feather pecking. The coarser components contained lower levels of nutrients important for preventing feather pecking (e.g., methionine, sodium). The study demonstrated that a feed structure that is too coarse can be considered a risk factor for feather pecking.

**Abstract:**

In this cross-sectional study, 103 complete feed samples from laying hen herds affected by plumage damage as an indirect measure for severe feather pecking (affected herds; AH, *n* = 37) and control herds without plumage damage (control herd; CH, *n* = 66) of commercial German farms were examined by dry sieve and nutrient analysis. AH showed higher percentages of particles >2.50 mm (mean ± SD, CH: 11.0 ± 8.5%, AH: 24.9 ± 14.3%) and 2.00–2.50 mm (CH: 11.2 ± 5.3%, AH: 15.7 ± 5.7%), but lower proportions of fractions 1.01–1.60 mm (CH: 22.9 ± 4.9%, AH: 17.8 ± 5.7%), 0.51–1.00 mm (CH: 25.5 ± 8.2%, AH: 16.0 ± 6.8%) and ≤0.50 mm (CH: 15.4 ± 5.0%, AH: 11.0 ± 4.8%) (*p* < 0.001). Diets of AH had a higher geometric mean diameter (GMD) compared to CH (AH: 1470.8 ± 343.9 μm; CH: 1113.3 ± 225.7 μm) (*p* < 0.001). Contents of crude ash (CH: 130.3 ± 18.8 g/kg, AH: 115.9 ± 24.3 g/kg), lysine (CH: 8.2 ± 1.0 g/kg, AH: 7.7 ± 1.2 g/kg), methionine (CH: 3.4 ± 0.5 g/kg, AH: 3.2 ± 0.6 g/kg) and sodium (CH: 1.7 ± 0.4 g/kg, AH: 1.3 ± 0.4 g/kg) were lower in AH (*p* ≤ 0.041). In a logistic regression model, animal age (*p* = 0.041) and GMD (*p* < 0.001) were significant factors on the occurrence of plumage damage.

## 1. Introduction

Severe feather pecking (SFP) and cannibalism in laying hens are undesirable allopecking behaviors that lead to plumage damage and skin/toe injuries, thus affecting animal welfare, performance, and profitability [1,2]. Within the multifactorial causes of SFP, animal nutrition plays a crucial role along with factors of husbandry, management, environmental enrichment, and genetics [3,4,5,6]. 

In addition to nutrient composition, feed structure is a major factor affecting digestibility, animal health, and performance of poultry [7,8]. Feed structure refers to the composition and size of the feed particles and the presentation of the compound feed (e.g., mash form, pellets) [9,10]. Given its importance to animal health and productivity, a balanced intake of structured feeds is considered important for optimal nutrition [11]. In this regard, chickens prefer larger feed particles [9,12]. Higher feed intake with coarser feed was observed in some reports [13], but not in all studies [7,14]. 

The effects of the feed structure on allopecking behaviors become evident when comparing pellets and feed in mash form. Pelleted feeds significantly increase the risk of SFP and plumage damage, especially when hens are housed in a low-stimulus environment [15]. With a finer grinding of mash form feeds, Walser [16] found a better plumage condition in hens, possibly caused by a prolonged feed intake time. Lieboldt et al. [17] demonstrated increased levels of coarse feed particles in the diet of feather-pecking laying hen flocks. Recently, relevant deviations from current particle size distribution recommendations for feed in mash form [18,19] in routinely sampled compound feed for laying hens were shown by Grünewald et al. [20]. Further risk factors for SFP related to nutrient composition of the diets include deficiencies in methionine and cysteine [21], lysine [22], sodium [23], magnesium [24], or crude fiber [25,26,27,28]. 

The relationship between the particle size distribution in the feed, the nutrient composition of the different particle size fractions and the occurrence of feather damage is not fully understood. Therefore, the following hypotheses were tested in this study: (1) a deviating particle size distribution in the feed is found in feather pecking laying flocks compared to unaffected flocks, and (2) the nutrient composition of the different particle size fractions differs with respect to ingredients that may be associated with SFP.

## 2. Materials and Methods

### 2.1. Herds and Integument Scoring

In total, 103 commercial laying hen herds from Germany were included in this study. All of the farms participated in a consulting project to optimize herd management and were managed non-organically with floor or free-range systems. Housing systems, hybrid types, feeding phases, feed supply sources, flock sizes and animal ages of the voluntarily participating flocks at the time of sampling are summarized in Table 1.

No experimental animals were used in the present study. It was part of a project funded and audited by the German Federal Institute of Food and Agriculture (grant number 2815HS004). Only data that have to be collected in production plants according to German legislation are evaluated in the study (i.e., observational study without any additional handling or treatment of the hens). The plumage condition of laying hens was investigated as an animal-associated trait, which poultry farms are obliged to collect according to the German Animal Welfare Act (§ 11 No. 8) [29] as part of the farm’s own inspection. In compliance with the European Directive 2010/63/EU [30], the experiment did not imply any invasive treatment of the hens. The animals were kept in accordance with the legal measures of the EU (Council Directive 1999/74/EC, minimum standards for the protection of laying hens) [31] and Germany (Animal Welfare Act [29]; Animal Protection Keeping of Production Animals Order [32]).

In the laying hen flocks, a survey of animal welfare indicators was carried out at 14-day intervals as part of the self-monitoring that is mandatory for livestock farmers in Germany according to §11 (8) of the German Animal Welfare Act [29]. The farmers performed plumage scoring using a 3-point-scale (score 0, feather-free areas < 1 cm; score 1, feather-free areas 1–5 cm; score 2, feather-free area > 5 cm) based on Welfare Quality^®^ [33] and modified according to Keppler [34] for back, belly (including cloacal region and ventral rump) and dorsal neck in a sample of 50 randomly selected hens per flock and survey time. This assessment was used to indirectly quantify the occurrence of SFP [1]. Based on the plumage condition determined at the time of feed sampling and the plumage condition determined 14 days earlier, flocks were divided into control herds (CH) and herds affected from increasing plumage damage (affected herds, AH). Flocks were defined as AH if (1) they had an increase of more than 10% in moderate plumage damage (score 1) and/or an increase of more than 5% in severe plumage damage (score 2) in one or more body regions between the last two assessments, or (2) at the time of sampling, more than 30% of the assessed hens had severe plumage damage in at least one of the scored regions. All other flocks were defined as CH (data not shown). Herds that showed relevant plumage damage (≥10% moderate plumage damage and/or ≥5% severe plumage damage) already at the first assessment after housing (i.e., poor pullet quality regarding plumage condition) were excluded from the study.

### 2.2. Feed Sampling and Analysis

A compound feed sample was collected from each of the enrolled flocks and analyzed for energy and nutrient contents and particle size distribution. The sampling times were chosen to represent three performance periods as described by Schreiter and Damme [35]: (1) from four weeks after housing (i.e., from week 21) to week 33 as a period of substantial body mass gain and increasing egg production, (2) weeks 34–52 as a period of increasing or maximum egg mass production, and (3) weeks 53–78 as a period of decreasing egg mass production. Therefore, herds were assigned to one of the three periods for feed sampling using random number generator in Microsoft Excel^®^ (version 2013, Microsoft Corporation, Redmond, WA, USA). Sampling was conducted according to the requirements of Commission Regulation (EC) No. 152/2009 [36]. All samples were subjected to nutrient and dry sieve analysis. To determine the nutrient content of each feed particle fraction, pool samples were formed for seven particle size fractions (≤0.50 mm, 0.51–1.00 mm, 1.01–1.60 mm, 1.61–2.00 mm, 2.01–2.50 mm, >2.50 mm) from eight individual feed samples each. For this purpose, four samples each from AH and CH were selected using the random number generator in Microsoft Excel^®^ (see above). Pool samples per particle fraction were formed by merging 10 g per fraction and individual sample.

Nutrient analysis of the samples was carried out at the laboratory of LUFA-ITL GmbH (Kiel/Germany) according to the current specifications of the Association of German Agricultural Analytic and Research Institutes [37] for chemical analyses of feed based on Commission Regulation (EC) No. 152/2009 [36]. Dry matter, crude protein, crude fat, crude ash, crude fiber, starch, sugar, calcium, sodium, magnesium, lysine, methionine (as methionine sulfone) and cysteine (as cysteic acid) were analyzed. Apparent metabolizable energy (AME_N_) was calculated based on the analyzed crude protein, crude fat, starch, and sugar contents using the World Poultry Science Association formula [38] according to Commission Regulation (EC) No. 152/2009 [36].

**Figure 1:**
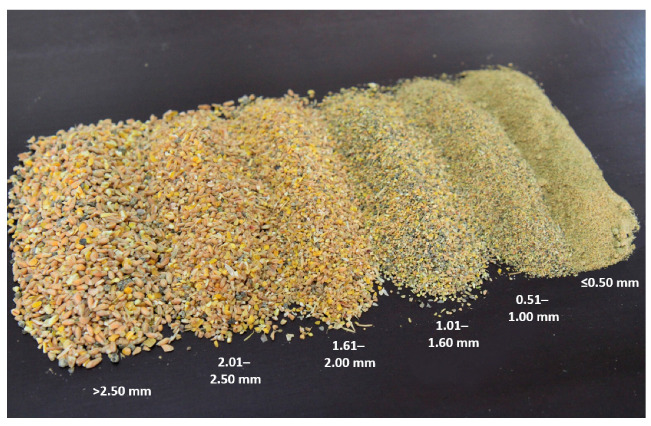
Feed particle fractions after a sieve analysis of a laying hen complete diet.

Dry sieve analysis was performed to determine particle size distribution [39,40]. A sample of 100 g was shaken for 10 min at an amplitude of 8 in a sieve tower (Retsch AS 200, Retsch GmbH, Haan, Germany) with seven sieves of mesh sizes of 3150, 2500, 2000, 1600, 1000, 500, and 250 μm (analysis sieves, Retsch GmbH, Haan, Germany). After each sieving, the retained feed on the sieves and the bottom was determined by a Navigator NV1101 digital scale (Ohaus Corporation, Parsippany, NJ, USA) for each mesh size. Each sieve with feed was weighed and the mass of the feed was determined by subtracting the mass of the empty sieve. To further characterize the particle sizes, the geometric mean diameter (GMD) as a single metrically scaled value and the geometric standard deviation (GSD) as a measure of variability for each sample were calculated [41]. In the data analysis and presentation of the results, the feed particle fractions were combined into the fractions of the current recommendations on feed structure for laying hens according to [19] (Figure 1). The relative proportions of the particle size fractions are given as mass fraction from the total mass (weight/weight).

### 2.3. Statistical Analyses

Microsoft Excel^®^ (Version 2013, Microsoft Corporation, Redmond, WA, USA) was used for data collection and processing. For further descriptive and inferential statistical analyses, the IBM SPSS Statistics program (Version 23, SPSS Inc., Chicago, IL, USA) was used. Diagrams were created in both software programs. For all metrically scaled traits under investigation, normal distribution of residuals and variance homogeneity were confirmed by the Kolmogorov–Smirnov test and Levene test, respectively [42].

In a first step, univariate statistical analyses were performed. For testing the effect of feed particle size on energy and nutrient contents, one-factorial linear ANOVA models [43] were used with the fixed effect of feed particle fraction. For post hoc pairwise comparisons, Hochberg’s GT2 (Generalized Tukey 2) test was applied [42,44]. Testing for differences between CH and AH in nutrient contents and mass proportions of each feed particle fraction was performed using Student’s t-test for independent samples [43]. The Benjamini–Hochberg procedure was employed to control the false discovery rate (FDR) due to multiple testing [45] by using an online tool [46]. 

For comparison of the proportion of the feed particle fractions with reference values for laying hens [19], one-sample t-tests were performed [42]. For this, the mean proportions of the respective feed particle fractions over all samples were compared with the minimum and maximum of the reference. A deviation of the proportion from the reference range was present if the t-test showed a significant difference from the reference minimum and maximum and the calculated confidence intervals of the mean of the samples did not overlap with the reference range [43].

In a second step, a binary logistic regression (BLR) model [47] was computed to evaluate the effect of the independent variables housing system (i.e., barn housing vs. free-range systems), hybrid type (i.e., brown egg layers vs. white egg layers), feed supply source (i.e., commercial compound feed plant vs. farm feed mixture), animal age at sampling, herd size (i.e., hens housed per herd) and GMD as well as the interaction GMD*hybrid type on the occurrence of plumage damage (i.e., herd classification as dependent variable, AH vs. CH). For the final model, independent variables and the interaction were kept when *p* < 0.10 in an attempt to reduce the type II error risk while maintaining a stringent type I error risk of 5%, using a backward selection approach [48]. The absence of multicollinearity was ensured by calculating Pearson correlation coefficients and using collinearity diagnostics with variance inflation factor and condition index [49,50]. In addition to GMD, other feed-associated traits (i.e., energy and nutrient contents) were not offered to the model due to their significant correlation with GMD. Nagelkerke’s R^2^ values, which give an indication of the extent of the variation of the dependent variables explained by the model, were calculated. Nagelkerke’s R^2^ values ≥0.5 were considered as very good and values in the range 0.4 ≤ R^2^ < 0.5 as good [51]. 

In all of the described inferential statistical analyses, differences were considered statistically significant for *p* ≤ 0.05 and tended to be significant if 0.05 < *p* ≤ 0.1.

## 3. Results

Significant differences between the particle size fractions were present for all analyzed traits of energy and nutrient contents (Table 2). Particles ≤1.00 mm had lower energy contents with higher levels of crude ash, crude fiber, lysine, methionine, calcium, sodium and magnesium compared to fractions >2.00 mm (*p* < 0.001). The highest contents of cysteine were found in the fraction 0.51–2.50 mm (*p* < 0.001), of crude protein in the fraction 0.51–1.00 mm (*p* ≤ 0.008) and of crude fat in the fraction ≤ 0.50 mm (*p* < 0.001). 

**Figure 2:**
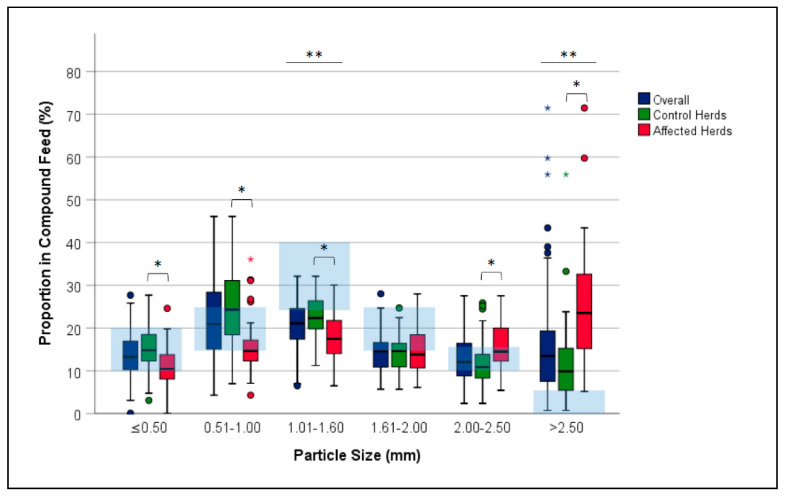
Box-whisker plots illustrating the proportions of feed particle fractions in compound feed samples (*n* = 103) from laying hen herds with and without behavioral disorders.* significant differences (*p* ≤ 0.05) in the proportions of the respective particle size fraction between control and affected herds; ** significant differences in the proportions of the respective particle size fraction between the compound feed samples (overall) and the reference range (light blue shaded) according to Pottgüter et al. [19].

When the feed samples of CH (*n* = 66) were compared with those of AH (*n* = 37), differences were found in both nutrient (*p* ≤ 0.036) and particle composition (*p* < 0.001). The levels of crude ash, lysine, methionine and sodium were lower in AH diets (Table 3). Feed samples from AH showed higher proportions of particles >2.50 mm (mean ± standard deviation, CH: 11.0 ± 8.5%, AH: 24.9 ± 14.3%; *p* < 0.001) and 2.00–2.50 mm (CH: 11.2 ± 5.3%, AH: 15.7 ± 5.7%; *p* < 0.001) at lower proportions of fractions 1.01–1.60 mm (CH: 22.9 ± 4.9%, AH: 17.8 ± 5.7%; *p* < 0.001), 0.51–1.00 mm (CH: 25.5 ± 8.2%, AH: 16.0 ± 6.8%; *p* < 0.001) and ≤0.50 mm (CH: 15.4 ± 5.0%, AH: 11.0 ± 4.8%; *p* < 0.001) (Figure 2). No difference was observed between AH and CH in the fraction 1.61–2.00 mm (CH: 14.0 ± 4.0%, AH: 14.5 ± 5.2%; *p* = 0.561). GMD was significantly different between CH (1113.3 ± 225.7 μm) and AH (1470.8 ± 343.9 μm) (*p* < 0.001). Regarding the homogeneity of the feed structure, there were no differences between AH (GSD: 2.06 ± 0.16 μm) and CH (2.01 ± 0.16 μm; *p* = 0.129). No differences existed in GMD between the three tested feeding phases (phase 1: 1214.8 ± 342.4 μm, phase 2: 1273.7 ± 276.1 μm, phase 3: 1313.3 ± 347.0 μm; *p* = 0.556).

Over all analyzed samples, there was a deviation from the reference values in the proportion of the feed particle fraction 1.01–1.60 mm (mean, 95% confidence interval: 21.1%, 20.0–22.2%; recommendation: 25.0–40.0%) (*p* < 0.001) and the fraction ≥ 2.50 mm (16.0%, 13.0–18.5%; recommendation: 0.0–5.0%) (*p* < 0.001). 

The significant final BLR model (*p* < 0.001) included animal age (*p* = 0.041), GMD (*p* < 0.001), and hybrid type (*p* = 0.067) as predictors of the occurrence of herds affected by plumage damage (Table 4). The Hosmer–Lemeshow test indicated validity (*p* = 0.416) and Nagelkerke’s R^2^ value of 0.473 showed a good explanatory quality of the model. The independent variables excluded from the final model were housing system (*p* = 0.714), feed supply source (*p* = 0.896), herd size (*p* = 0.801), and GMD*hybrid type interaction (*p* = 0.364). 

## 4. Discussion

The objectives of this study were to identify a possible relationship between feed structure and the occurrence of plumage damage and to compare the nutrient composition of relevant feed particle fractions. As indicated in previous studies [17,52], our field study showed a clear effect of feed structure on the occurrence of plumage damage on commercial farms. The further effects of animal age and hybrid type on plumage condition are already known [53].

The fact that the feed particle fractions differed in their energy, nutrient, and active ingredient contents is an expected result, without any reliable values being available so far. Since the coarse particles are primarily more or less crushed cereal grains [17], high energy at low mineral and amino acid contents is conclusive. Over all samples, the observed fraction >2.50 mm had a mass proportion of 16.0% and was beyond the reference range of 0.0–5.0% as recommended by [19]. Thus, the grain used in the feed often has an insufficient degree of comminution. The degree of grinding depends in particular on the mill type and technique of grinding (e.g., hammer mill vs. roller mill), but also on the quality of the components (e.g., plant-anatomical effects) [7,35]. There is evidence that coarser grinding improves starch digestibility [7] and has positive effects on weight and activity of the gizzard [39,54].

Three approaches should be considered as possible causes for the higher risk of plumage damage with higher GMD of feed particles. First, a coarser feed structure is associated with a shorter time for feed intake compared to finely ground feed [16]. Since SFP is considered to be misdirected pecking as a result of deficits in foraging and feed intake behavior [55,56,57], prolonged engagement with more pecking in the feed can be realized by smaller feed particles. Second, inhomogeneous feed mixtures with high levels of coarse particles promote selective intake of coarser feed components, resulting in an imbalanced nutrient supply [17]. Given the low levels of methionine and sodium found in feed particles >2.00 mm and >1.60 mm, respectively, and the known response of hens with SFP to a deficiency of methionine [21] and sodium [23], this effect seems remarkable. Third, effects on feed intake, digestive organ development, and nutrient absorption associated with variations in feed particle size [8,9] may further show an effect on the occurrence of plumage damage.

Levels of methionine and lysine play an important role in the risk of feather pecking [15]. Both amino acid contents were significantly lower in the diets of the AH than in CH. In addition, a higher proportion of coarse particles was found in the feed of AH. These coarse particles may force selective feeding and had lower lysine and methionine contents than finer particles. Schreiter and Damme [35] identified the problem of selective feed intake especially in flat chain feeding systems in barns with a long longitudinal axis. In a barn with a length of 90 m and three consecutive compartments, the authors found that particles > 2 mm were already eaten predominantly by the hens in the first compartment when the feed chain was running, thus promoting an unbalanced nutrient supply. Therefore, in addition to adjusting the grind of the mash feed, other technical measures of food provision to reduce selective feed intake appear reasonable. For example, Pottgüter et al. [19] recommend regular empty feeding of the feed troughs, longer intervals between feedings, and a high speed of the feed chains. In the case of higher proportions of coarse grain particles in the feed, the administration of hydrochloric acid-insoluble grit stones is also beneficial to promote mechanical comminution of the feed components in the gizzard [35].

In this study, a relation between feed particle sizes and feather condition on laying hen farms was shown. It seems necessary to investigate the influence of feed structure on the occurrence of feather pecking in more detail in follow-up studies with a longitudinal study design over the entire laying period. In addition, the feed structure in pullet rearing and possible effects in the later laying phase should also be examined, especially from the view that changes in the feed structure can occur between rearing and laying feeds. Due to possible genotype–environment interactions, as recently observed in the provision of edible enrichment materials [58], different hybrid strains should be included in the study. In long production barns, a possible interaction between compartment or location on the forward or return side of the feeding chain and GMD in the effect on integument condition should be investigated. In addition, it should be clarified whether the effects of feed structure are also present in other housing systems (organic housing, small group housing, cage systems). Furthermore, an assessment of inter- and intra-observer reliability for the integument scoring, which was not possible in this study, is recommended in follow-up studies to avoid bias.

## 5. Conclusions

A relation between feed structure and the occurrence of plumage damage in the field was demonstrated. The different nutrient contents of the individual particle size fractions are of crucial importance for this relationship. Highly inhomogeneous complete feed mixtures in mash form with increased proportions of particles >2.00 mm represent a risk factor for the occurrence plumage damage in laying hens. We therefore recommend that laying hen farmers regularly check the feed particle fractions by sieve analysis. In further studies with a longitudinal design over the entire laying period, the influence of feed structure on the behavior of laying hens should be investigated. 

## Figures and Tables

**Table 1:** Housing system, hybrid type, feed supply source, age of hens, and herd size of the laying hen herds (*n* = 103).

Trait	Unit	Descriptive Statistics		
		Total	Control Herds	Affected Herds
Housing system				
Barn housing	*n* (%)	65 (63.1)	40 (60.6)	25 (67.6)
Free-range system	*n* (%)	38 (36.9)	26 (39.4)	12 (32.4)
Hybrid type				
Brown-egg layers	*n* (%)	75 (72.8)	44 (66.7)	31 (83.8)
White-egg layers	*n* (%)	28 (27.2)	22 (33.3)	6 (16.2)
Feeding phase				
Phase 1	*n* (%)	62 (60.2)	46 (69.7)	16 (43.2)
Phase 2	*n* (%)	32 (31.1)	18 (27.3)	14 (37.8)
Phase 3	*n* (%)	9 (8.7)	2 (3.0)	7 (18.9)
Feed supply source				
Commercial compound feed plant	*n* (%)	91 (88.3)	58 (87.9)	33 (89.2)
Farm feed mixture	*n* (%)	12 (11.7)	8 (12.1)	4 (10.8)
Age of the hens (weeks)	mean ± SD ^1^	43.9 ± 15.3	40.2 ± 13.9	50.7 ± 15.5
Hens housed per herd	median (1.–3. quartile)	5970 (2040–14,954)	5870 (1854–14,771)	6350 (2995–16,291)

^1^ mean—arithmetic mean, SD—standard deviation.

**Table 2:** Energy and nutrient contents in different particle size fractions in compound feeds (*n* = 8) for laying hens.

Content	Unit ^1^	Particle Size Fraction ^2^ (Mean ± SD ^3^)						*p*-Value
		≤0.50 mm	0.51–1.00 mm	1.01–1.60 mm	1.61–2.00 mm	2.01–2.50 mm	>2.50 mm	
Dry matter	g/kg	907.2 ± 35.6	900.7 ± 35.4	906.5 ± 35.6	884.1 ± 34.7	880.6 ± 34.6	876.4 ± 34.4	0.299
AME_N_^4^	MJ/kg	10.3 ± 0.4 ^a^	10.0 ± 0.4 ^a^	10.0 ± 0.4 ^a^	12.2 ± 0.5 ^b^	12.4 ± 0.5 ^b^	12.5 ± 0.5 ^b^	<0.001
Crude protein	g/kg	134.1 ± 5.3 ^a^	193.7 ± 7.6 ^e^	181.6 ± 7.1 ^d^	172.4 ± 6.8 ^c^	147.3 ± 5.8 ^b^	133.4 ± 5.2 ^a^	<0.001
Crude ash	g/kg	218.8 ± 8.6 ^e^	181.7 ± 7.1 ^c^	197.4 ± 7.8 ^d^	82.3 ± 3.2 ^b^	77.2 ± 3.0 ^b^	57.7 ± 2.3 ^a^	<0.001
Crude fat	g/kg	68.1 ± 2.7 ^e^	53.2 ± 2.1 ^d^	41.7 ± 1.6 ^ab^	41.8 ± 1.6 ^b^	39.2 ± 1.5 ^a^	46.6 ± 1.8 ^c^	<0.001
Crude fiber	g/kg	32.1 ± 1.3 ^b^	38.8 ± 1.5 ^d^	34.1 ± 1.3 ^c^	31.7 ± 1.2 ^b^	27.8 ± 1.1 ^a^	29.3 ± 1.2 ^a^	<0.001
Lysine	g/kg	7.2 ± 0.3 ^c^	10.3 ± 0.4 ^d^	9.4 ± 0.4 ^d^	7.7 ± 0.3 ^c^	6.0 ± 0.2 ^b^	5.0 ± 0.2 ^a^	<0.001
Methionine	g/kg	7.1 ± 0.3 ^d^	4.3 ± 0.2 ^c^	3.0 ± 0.1 ^b^	2.8 ± 0.1 ^b^	2.4 ± 0.1 ^a^	2.2 ± 0.1 ^a^	<0.001
Cysteine	g/kg	2.1 ± 0.1 ^a^	2.8 ± 0.1 ^c^	2.9 ± 0.1 ^c^	2.9 ± 0.1 ^c^	2.8 ± 0.1 ^c^	2.5 ± 0.1 ^b^	<0.001
Calcium	g/kg	70.3 ± 2.8 ^e^	61.8 ± 2.4 ^c^	67.5 ± 2.7 ^d^	21.3 ± 0.8 ^b^	23.1 ± 0.9 ^b^	14.7 ± 0.6 ^a^	<0.001
Sodium	g/kg	4.5 ± 0.2 ^d^	2.7 ± 0.1 ^c^	1.0 ± 0.1 ^b^	0.3 ± 0.1 ^a^	0.2 ± 0.1 ^a^	0.2 ± 0.1 ^a^	<0.001
Magnesium	g/kg	3.1 ± 0.1 ^d^	2.5 ± 0.1 ^c^	2.6 ± 0.1 ^c^	1.6 ± 0.1 ^b^	1.4 ± 0.1 ^ab^	1.3 ± 0.1 ^a^	<0.001

^1^ as feed. ^2^ different indices indicate significant differences. ^3^ mean—arithmetic mean, SD—standard deviation. ^4^ apparent metabolizable energy. *n*-corrected.

**Table 3:** Energy and nutrient contents in compound feed samples from laying hen herds with (*n* = 37) and without plumage damage (*n* = 66).

Content	Unit ^1^	Group (Mean ± SD ^2^)			*p*-Value
		All Samples	Control Herds	Affected Herds	
Dry matter	g/kg	883.6 ± 28.1	885.4 ± 26.2	880.7 ± 34.3	0.828
AME_N_^3^	MJ/kg	11.1 ± 0.5	11.0 ± 0.5	11.1 ± 0.6	0.424
Crude protein	g/kg	166.1 ± 13.0	166.8 ± 12.4	164.8 ± 13.9	0.474
Crude ash	g/kg	125.1 ± 21.9	130.3 ± 18.8	115.9 ± 24.3	0.006
Crude fat	g/kg	49.2 ± 11.2	49.6 ± 11.9	48.5 ± 9.9	0.657
Crude fiber	g/kg	40.9 ± 13.3	42.3 ± 14.8	38.4 ± 12.4	0.279
Lysine	g/kg	8.0 ± 1.1	8.2 ± 1.0	7.7 ± 1.2	0.041
Methionine	g/kg	3.3 ± 0.5	3.4 ± 0.5	3.2 ± 0.6	0.029
Cysteine	g/kg	2.9 ± 0.2	2.9 ± 0.2	2.9 ± 0.3	0.344
Calcium	g/kg	36.2 ± 9.0	37.3 ± 8.7	34.3 ± 9.4	0.266
Sodium	g/kg	1.5 ± 0.4	1.7 ± 0.4	1.3 ± 0.4	<0.001
Magnesium	g/kg	1.9 ± 0.4	1.9 ± 0.3	2.0 ± 0.6	0.356

^1^ as feed. ^2^ mean—arithmetic mean, SD—standard deviation. ^3^ apparent metabolizable energy. N-corrected.

**Table 4:** Effects of hybrid type, age and geometric mean diameter of feed particles on the occurrence of plumage damage in laying hen herds—results of a logistic regression model.

Trait	Coefficients (SE)	Odds Ratio (95% CI)	*p*-Value
Hybrid type			
brown-egg layers	reference	baseline	
white-egg layers	−1.140 (0.622)	0.320 (0.095–1.082)	0.067
age	0.038 (0.019)	1.039 (1.002–1.078)	0.041
GMD	0.005 (0.001)	1.005 (1.003–1.007)	<0.001
constant	−8.369 (1.708)		

SE—standard error. CI—confidence interval. GMD—Geometric Mean Diameter.

## Data Availability

The data presented in this study are available on request from the corresponding author. The data are not publicly available due to privacy reasons.

## References

[1] Bilcik B., Keeling L.J. (1999). Changes in feather condition in relation to feather pecking and aggressive behaviour in laying hens. Br. Poult. Sci..

[2] Rodenburg T.B., van Krimpen M.M., de Jong I.C., de Haas E.N., Kops M.S., Riedstra B.J., Nordquist R.E., Wagenaar J.P., Bestman M., Nicol C.J. (2013). The prevention and control of feather pecking in laying hens: Identifying the underlying principles. World’s Poult. Sci. J..

[3] Klein T., Zeltner E., Huber-Eicher B. (2000). Are genetic differences in foraging behaviour of laying hen chicks paralleled by hybrid-specific differences in feather pecking?. Appl. Anim. Behav. Sci..

[4] Van Krimpen M., Kwakkel R., Reuvekamp B., van der Peet-Schwering C., den Hartog L., Verstegen M. (2005). Impact of feeding management on feather pecking in laying hens. World’s Poult. Sci. J..

[5] Schreiter R., Damme K., von Borell E., Vogt I., Klunker M., Freick M. (2019). Effects of litter and additional enrichment elements on the occurrence of feather pecking in pullets and laying hens—A focused review. Vet. Med. Sci..

[6] Schreiter R., Damme K., Klunker M., Raoult C., von Borell E., Freick M. (2020). Effects of edible environmental enrichments during the rearing and laying periods in a littered aviary—Part 1: Integument condition in pullets and laying hens. Poult. Sci..

[7] Ruhnke I., Röhe I., Krämer C., Goodarzi Boroojeni F., Knorr F., Mader A., Schulze E., Hafeez A., Neumann K., Löwe R. (2015). The Effects of Particle Sizes, Milling Methods, and Thermal Treatment of Feed on Performance, Apparent Ileal Digestibility and pH of the Digesta in Laying Hens. Poult. Sci..

[8] Bozkurt M., Gianneııas I., Cabıık M., Tüzin A.E. (2020). The effect of feed structure on gastrointestinal tract traits and performance in laying hens: An overview of 70 years’ experience. World’s Poult. Sci. J..

[9] Amerah A.M., Ravindran V., Lentle R.G., Thomas D.G. (2007). Influence of Feed Particle Size and Feed Form on the Performance, Energy Utilization, Digestive Tract Development and Digesta Parameters of Broiler Starters. Poult. Sci..

[10] Svihus B. (2014). Function of the Digestive System. J. Appl. Poult. Res..

[11] Yegani M., Korver D.R. (2008). Factors affecting intestinal health in poultry. Poult. Sci..

[12] Yasar S. (2003). Performance, gut size and ileal digesta viscosity of broiler chickens fed with a whole wheat added diet and diets with different wheat particle sizes. Int. J. Poult. Sci..

[13] Safaa H.M., Jimenez-Moreno E., Valencia D.G., Frikha M., Serrano M.P., Matheos G.G. (2009). Effect of main cereal of the diet and particle size of the cereal on productive performance and egg quality of brown egg-laying hens in early phase of production. Poult. Sci..

[14] Kocer B., Bozkurt M., Kücükyilmaz K., Ege G., Aksit H., Orojpour A., Topbas S., Tüzün A.E., Bintas E., Seyrek K. (2016). Effects of particle sizes and physical form of the diet on performance, egg quality and size of the digestive organs in laying hens. Europ. Poult. Sci..

[15] Kjaer J.B., Bessei W. (2013). The interrelationships of nutrition and feather pecking in the domestic fowl—A review. Arch. Geflügelk..

[16] Walser P.T. (1997). Einfluss Unterschiedlicher Futterzusammensetzung und -Aufarbeitung auf das Auftreten von Federpicken, das Nahrungsaufnahmeverhalten, die Leistung und den Gesamtstoffwechsel bei Verschiedenen Legehennenhybriden. Ph.D. Thesis.

[17] Lieboldt M.A., Borgelt L., Wolf P. (2018). Mischfuttermittel für Legehennen—Auf Spurensuche mittels Siebanalyse. DGS-Magazin.

[18] Lohmann Breeders GmbH (2020). Management Guide. https://lohmann-breeders.com/media/strains/cage/management/LOHMANN-Brown-Classic-Cage.pdf.

[19] Pottgüter R., Schreiter R., van der Linde J., Damme K., Mayer A. (2018). Management recommendations for rearing and husbandry of laying hens in floor, aviary and free range systems. Poultry Annual 2019.

[20] Grünewald K.H., Damme K., Schwick S. (2019). Rohfasergehalt und Partikelgrößenverteilung im Mischfutter für Legehennen. VDLUFA-Schr. Kongr..

[21] Hughes B.O., Duncan I.J.H. (1972). The influence of strain and environmental factors upon feather picking and cannibalism in fowls. Br. Poult. Sci..

[22] Conson M., Petersen J. (1986). Beurteilung der Gefiederbeschaffenheit unterschiedlich aufgezogener Legehybriden. Arch. Geflügelk..

[23] Cooke B.C. (1992). Cannibalism in laying hens. Vet. Rec..

[24] Schaible P.J., Davidson A., Bandemer S.L. (1947). Cannibalism and feather pecking in chicks as influenced by certain changes in a specific ration. Poult. Sci..

[25] Hartini S., Choct M., Hinch G., Kocher A., Nolan J.W. (2002). Effects of light intensity during rearing and beak trimming and dietary fiber sources on mortality, egg production, and performance of ISA Brown laying hens. J. Appl. Poult. Res..

[26] Van Krimpen M.M., Kwakkel R.P., van der Peet-Schwering C.M., den Hartog L.A., Verstegen M.W. (2008). Low dietary energy concentration, high nonstarch polysaccharide concentration, and coarse particle sizes of nonstarch polysaccharides affect the behavior of feather-pecking-prone laying hens. Poult. Sci..

[27] Qaisrani S.N., van Krimpen M.M., Kwakkel R.P. (2013). Effects of dietary dilution source and dilution level on feather damage, performance, behavior, and litter condition in pullets. Poult. Sci..

[28] Schreiter R., Damme K., Hartmann J., Klunker M., Freick M., Wolff N., von Borell E. (2019). Effect of a specially to reduce feather pecking designed feed on the performance and the occurrence of behavioural disorders in laying hens. Europ. Poult. Sci..

[29] TierSchG Tierschutzgesetz in der Fassung der Bekanntmachung vom 18. Mai 2006 (BGBl. I S. 1206, 1313), das Zuletzt Durch Artikel 280 der Verordnung vom 19. Juni 2020 (BGBl. I S. 1328) Geändert Worden ist. http://www.gesetze-im-internet.de/tierschg/TierSchG.pdf.

[30] (2010). Council Directive 2010/63/EU of the European Parliament and of the Council of 22 September 2010 on the Protection of Animals Used for Scientific Purposes. Off. J..

[31] Council Directive 1999/74/EC of 19 July 1999 Laying down Minimum Standards for the Protection of Laying Hens. http://www.legislation.gov.uk/eudr/1999/74/contents.

[32] TierSchNutztV Verordnung zum Schutz Landwirtschaftlicher Nutztiere und Anderer zur Erzeugung Tierischer Produkte Gehaltener Tiere bei Ihrer Haltung.—Tierschutz-Nutztierhaltungsverordnung in der Fassung der Bekanntmachung vom 22. August 2006, BGBl.I, 2043, die Durch Artikel 3 Absatz 2 des Gesetzes vom 30. Juni 2017 (BGBL I, 2147) Geändert worden ist. http://www.gesetze-iminternet.de/tierschnutztv/.

[33] Welfare Quality® (2009). Welfare Quality® Assessment Protocol for Poultry (Broilers, Laying Hens).

[34] Keppler C. (2017). Managementtool Beurteilungskarten—Legehennen. Anleitung zur Beurteilung des Tierzustandes. University Kassel. https://www.mud-tierschutz.de/fileadmin/user_upload/2017-08-22_Beurteilungskarten_Legehennen_web.pdf.

[35] Schreiter R., Damme K. (2017). Nutrition of Laying Hens—Use of Domestic Feed and Feeding of Laying Hens with Untrimmed Beaks.

[36] European Commission Commission Regulation (EC) No. 152/2009 of 27 January 2009 Laying Down the Methods of Sampling and Analysis for the Official Control of Feed. https://eur-lex.europa.eu/legal-content/EN/TXT/PDF/?uri=CELEX:32009R0152&from=de.

[37] Naumann C., Bassler R. (2004). Chemical analyses of feedstuff. Method book III.

[38] WPSA (World´s Poultry Science Association) (1984). The prediction of apparent metabolizable energy values for poultry in compound feeds. World’s Poult. Sci. J..

[39] Röhe I., Ruhnke I., Knorr F., Mader A., Goodarzi Boroojeni F., Löwe R., Zentek J. (2014). Effects of Grinding Method, Particle Size, and Physical Form of the Diet on Gastrointestinal Morphology and Jejunal Glucose Transport in Laying Hens. Poult. Sci..

[40] Ege G., Bozkurt M., Kocer B., Tüzün A.E., Uygun M., Alkan G. (2019). Influence of feed particle size and feed form on productive performance, egg quality, gastrointestinal tract traits, digestive enzymes, intestinal morphology, and nutrient digestibility of laying hens reared in enriched cages. Poult. Sci..

[41] Wolf P., Arlinghaus M., Kamphues J., Sauer N., Mosenthin R. (2012). Impact of feed particle size on nutrition digestibility and performance in pigs. Ubers. Tierern..

[42] Weiß C. (1999). Basic Knowledge of Medical Statistics.

[43] du Prel J.-B., Röhrig B., Hommel G., Blettner M. (2010). Auswahl statistischer Testverfahren. Dtsch. Aerztebl. Int..

[44] Rasch B., Friese M., Hofmann W.J., Naumann E. (2010). Quantitative Methods—Volume 2.

[45] Victor A., Elsäßer A., Hommel G., Blettner M. (2010). Wie bewertet man die p-Wert-Flut?. Dtsch. Aerztebl. Int..

[46] SDM Project Web—FDR Online Calculator. https://www.sdmproject.com/utilities/?show=FDR.

[47] Baltes-Götz B. (2000). Logistische Regressionsanalyse Mit SPSS. https://www.uni-trier.de/fileadmin/urt/doku/logist/logist.pdf.

[48] Hosmer D.W., Lemeshow S. (2000). Applied Logistic Regression.

[49] Menard S. (2002). Applied Logistic Regression Analysis.

[50] Field A. (2013). Discovering Statistics Using IBM SPSS Statistics.

[51] Backhaus K., Erichson B., Plinke W., Weiber R. (2016). Multivariate Analysemethoden.

[52] Velik M., Baumung R., Zaludik K., Niebuhr K., Zollitsch W., Heß J., Rahmann G. (2005). Feldstudie zu Futtereigenschaften bei federpickenden Legehennen. Ende der Nische, Beiträge zur 8. Wissenschaftstagung Ökologischer Landbau in Kassel.

[53] Spindler B., Giersberg M.F., Andersson R., Kemper N. (2016). Keeping laying hens with untrimmed beaks—A Review of the status quo in practice and science. Züchtungskunde.

[54] Roche M. (1981). Feeding behaviour and digestive motility of birds. Reprod. Nutr. Dev..

[55] Wennrich G. (1974). Ethological studies of domestic chickens (gallus domesticus) of different hybrid origin in floor management with special reference to aggressive behaviour as well as feather pecking and cannibalism. 1st Communication: Pecking movements in feeding behaviour. Arch. Geflügelk..

[56] Blokhuis H.J.J. (1986). Feather-pecking in poultry: Its relation with ground-pecking. Appl. Anim. Behav. Sci..

[57] Weeks C.A., Nicol C.J. (2006). Behavioural needs, priorities and preferences of laying hens. Worlds Poult. Sci. J..

[58] Schreiter R., Damme K., Freick M. (2020). Edible Environmental Enrichments in Littered Housing Systems: Do Their Effects on Integument Condition Differ Between Commercial Laying Hen Strains?. Animals.

